# La Douleur Centrale Post Accident Vasculaire Cérébral au Chu Tingandogo de Ouagadougou (Burkina Faso): Fréquence, Profil Clinique, Qualité de Vie des Patients et Facteurs Associés

**DOI:** 10.48327/1160-X245

**Published:** 2021-02-01

**Authors:** L.D. Lompo, A.M. Ouédraogo, A. Somé, O. Diallo, C. Napon, B.J. Kaboré

**Affiliations:** 1 CHU de Tingandogo, Unité de formation et de recherches des sciences de la santé, Université Ouaga I-Pr Joseph Ki-Zerbo, Ouagadougou, Burkina Faso; 2 CHU Yalgado Ouédraogo de Ouagadougou, Unité de formation et de recherches des sciences de la santé, Université Ouaga I-Pr Joseph Ki-Zerbo, Ouagadougou, Burkina Faso; 3 Institut de recherche en sciences de la sante, Ouagadougou, département biologie médicale et sante publique, Ouagadougou, Burkina Faso

**Keywords:** Douleur centrale post accident vasculaire cérébral, Qualité de vie, Age, Thérapeutique, Hôpital, Ouagadougou, Burkina Faso, Afrique intertropicale, Central post-stroke pain, Quality of life, Age, Therapeutics, Hospital, Ouagadougou, Burkina Faso, Sub-Saharan Africa

## Abstract

**Objectifs:**

Le but de la présente étude était de déterminer la fréquence de la douleur centrale post accident vasculaire cérébral (DCPA), de décrire son profil clinique, d'évaluer la qualité de vie des patients et d'identifier les facteurs associés à sa survenue, à partir d'une série hospitalière, prospective, à Ouagadougou, au Burkina Faso.

**Méthodologie:**

Il s'agissait d'une étude prospective de suivi longitudinal, descriptive et analytique, menée de janvier 2015 à mars 2020, au CHU de Tingandogo, à Ouagadougou, au Burkina Faso. Elle a concerné tous les patients âgés de plus de 16 ans, hospitalisés pour AVC confirmé par la TDM et/ou l'IRM encéphalique, puis revus tous les trois mois en consultation externe de neurologie et ce pendant au moins 9 mois après leur AVC. Les caractéristiques sociodémographiques et cliniques des patients, la nature de l'AVC, l'existence de DCPA et le cas échéant, ses caractéristiques cliniques, son traitement et son impact sur la qualité de vie des patients ont été relevés; une analyse bivariée puis multivariée avec régression logistique selon le modèle des risques proportionnels de Cox a permis de rechercher les facteurs associés à la survenue de la DCPA. Le seuil de significativité retenu a été de p < 0,05.

**Résultats:**

236 patients ont été colligés parmi lesquels 28 patients ont présenté une DCPA (11,9%), après une durée moyenne de suivi post AVC de 12,9 mois. L'infarctus cérébral, l'hémorragie intracérébrale et la thrombose veineuse cérébrale représentaient respectivement 69,5%, 29,7% et 0,8%. La moyenne d'âge des patients avec DCPA était de 54,6 ans, avec une prédominance masculine (53,6%). Le délai moyen d'apparition de la DCPA était de 3,8 mois après l'AVC. Les douleurs à type de brûlure (75%) et d'allodynies (67,8%), étaient les plus fréquentes. L'intensité moyenne de la DCPA était de 7,6/10 sur l'échelle visuelle analogique. Une hypoesthésie (96,4%) et des paresthésies (71,4%) étaient les signes ou symptômes les plus fréquemment associés à la DCPA. La DCPA avait un retentissement négatif modéré à sévère sur le travail habituel, l'activité générale et l'humeur des patients, respectivement chez 60,7%, 50% et 46,4% des patients. L'amitriptyline (75%) et/ou les antalgiques de palier II (60,7%) ont été les molécules les plus utilisées, et efficaces chez 57% des cas. Seul l'âge ≤ 50 ans était indépendamment associé à la survenue de la DCPA (OR 2, 86; p = 0,03).

**Conclusion:**

La DCPA touche plus d'un patient sur 10 victimes d'AVC et altère de façon modérée à sévère la qualité de vie de la plupart de ces patients. Le dépistage et la prise en charge adéquate de la DCPA dans le cadre du suivi pluridisciplinaire post AVC, contribueront à l'amélioration de la qualité de vie des patients victimes d'AVC et faciliteront leur réinsertion socioprofessionnelle.

## Abstract (en)

**Objective:**

Few studies have been done on central post-stroke pain (CPSP) in Sub-Saharan Africa, while taking it into account would improve the quality of life of stroke survivors. The purpose of this study was to determine the prevalence of CPSP, to describe its clinical profile, to assess the quality of life of patients and to identify the factors associated with its occurrence, from a prospective hospital series in Ouagadougou, Burkina Faso.

**Methodology:**

It was a prospective, descriptive and analytical longitudinal follow-up study, conducted from January 2015 to March 2020, at the Tingandogo University Hospital, in Ouagadougou, Burkina Faso. The study involved all patients over the age of 16, consecutively hospitalized for stroke confirmed by CT and / or brain MRI, then reviewed every three months in outpatient Neurology, during at least 9 months after their stroke. The sociodemographic and clinical characteristics of the patients, the nature of the stroke, the existence of CPSP and, if applicable, its clinical characteristics, its treatment and its impact on the quality of life of the patients were recorded; a bivariate then multivariate analysis with logistic regression step by step, made it possible to search for the factors associated with the occurrence of CPSP. The significance threshold used was p < 0.05.

**Results:**

A total of 236 patients were collected, out of which 28 patients presented a CPSP (11.9%), after a mean duration of post-stroke follow-up of 12.9 months. Cerebral infarction, intracerebral hemorrhage and cerebral venous thrombosis accounted for 69.5%, 29.7% and 0.8% respectively. The mean age of patients with CPSP was 54.6 years, with a male predominance (53.6%). The mean time to onset for CPSP was 3.8 months after stroke. Pains such as burning (75%) and allodynia (67.8%) were the most common. The average CPSP intensity was 7.6 / 10 on the visual analog scale. Hypoaesthesia (96.4%) and paraesthesia (71.4%) were the signs or symptoms most commonly associated with CPSP. CPSP had a moderate to severe negative impact on usual work, general activity and mood of patients in 60.7%, 50% and 46.4% of patients, respectively. Amitriptyline (75%) and / or level II analgesics (60.7%,) were the most used molecules, and effective in 57% of cases. Only age ≤ 50 years was independently associated with the occurrence of CPSP (OR 2.86; p = 0.03).

**Conclusion:**

CPSP affects more than 1 in 10 stroke patients and moderately to severely affects the quality of life for most of these patients. Screening and adequate management of CPSP as part of multidisciplinary post-stroke follow-up will contribute to improve the quality of life of stroke patients and will facilitate their social and professional reintegration.

## Introduction

La douleur centrale post accident vasculaire cérébral (DCPA) est un syndrome douloureux neuropathique, caractérisé par des douleurs constantes ou intermittentes dans une partie du corps, associé à des anomalies somatosensitives dans la partie douloureuse du corps, survenant comme une conséquence directe d'une lésion cérébrovasculaire [7, 10]. La DCPA avait été d'abord attribuée à une lésion thalamique et connue sous le nom de « syndrome de Dejerine et Roussy », mais elle est désormais également associée à des lésions extra-thalamiques, car ses symptômes et sa sévérité ne diffèrent pas dans les AVC thalamiques ou extra-thalamiques [35]. La DCPA survient après des lésions à n'importe quel niveau la voie spino-thalamo-corticale, ou au niveau du cortex insulaire ou operculaire [13]. Plusieurs mécanismes physiopathologiques sont impliqués dans la DCPA et comprennent: un déséquilibre entre le tractus spino-thalamique latéral impliqué dans la sensation de froid et les voies de signalisation de la douleur médiale [31]; un déséquilibre entre les impulsions facilitatrices et inhibitrices dans le système nerveux central [5]; des lésions des voies spino-thalamiques qui peuvent entraîner la transmission d'impulsions nociceptives par des voies alternatives – voies paléo-spino-thalamiques multisynaptiques [24].

Les AVC représentent une cause majeure de décès, de handicap acquis et de démence à travers le monde et en particulier dans les pays en développement [40]. La DCPA demeure encore de nos jours une complication peu connue et sous-estimée de l'AVC. Sa prévalence varie dans l'ensemble de 1 à 12% [24, 37], du fait des difficultés diagnostiques de ce syndrome en l'absence de critères diagnostiques pathognomoniques, de la variabilité des délais d'apparition de ce syndrome après l'AVC [24, 37]. Aucun traitement standard de la DCPA n'a encore été établi [43]: la DCPA est connue pour être résistante aux analgésiques conventionnels [40]. Même si une grande variété d'options de traitement a été suggérée, aucune règle universellement applicable n'est encore admise à ce jour [37].

La DCPA altère la qualité de vie des patients: elle perturbe la réadaptation fonctionnelle, le sommeil, conduit parfois à la dépression, à l'anxiété, à la toxicomanie et réduit les interactions sociales [21, 24, 41] et ses effets sur le handicap lié au déficit neurologique séquellaire de l'AVC [31].

Cependant, peu d'études ont été consacrées à la DCPA en Afrique sub-saharienne. À notre connaissance, 2 études réalisées au Nigeria ont retrouvé des prévalences respectives de 5% et 8%, une douleur apparaissant majoritairement dans les 3 premiers mois post AVC, une intensité de la douleur le plus souvent modérée et à type de brûlure [2], une DCPA ayant un impact significativement négatif sur la qualité de vie des patients [38].

La connaissance de la prévalence et du profil clinique de la DCPA et une estimation de son impact sur la qualité de vie des survivants d'AVC, permettraient de mieux planifier, organiser les soins et assurer une meilleure prise en charge des patients après AVC, contribuant à terme à une amélioration significative de la qualité de vie des patients survivants à l'AVC.

Les objectifs de la présente étude étaient ainsi de déterminer la prévalence de la DCPA, de décrire son profil clinique et thérapeutique, d'évaluer son impact sur la qualité de vie des patients et d'identifier des facteurs associés à sa survenue, à travers une étude prospective descriptive d'une cohorte de patients hospitalisés pour AVC puis suivis en consultation externe de neurologie dans le cadre du suivi post AVC, au CHU de Tingandogo, à Ouagadougou, au Burkina Faso.

## Patients et Méthodes

Il s'agissait d'une étude prospective de suivi longitudinal, descriptive et analytique, menée du 1^er^ janvier 2015 au 31 mars 2018 (39 mois) dans le service de neurologie du CHU de Tingandogo, à Ouagadougou, au Burkina Faso.

La population d'étude était constituée de tous les patients, âgés de plus de 16 ans, sans distinction de genre, consécutivement hospitalisés pour infarctus cérébral ou hémorragie intra cérébrale, confirmé par la neuro-imagerie (TDM et/ou IRM encéphalique), dans le dit service, durant la période d'étude. Les survivants parmi ces patients étaient ensuite revus en consultation externe de neurologie, dans le cadre d'un suivi le plus souvent trimestriel post AVC. Ces patients devaient avoir donné leur consentement éclairé pour participer à l'étude et un délai de suivi post AVC d'au moins 9 mois était nécessaire pour chaque patient inclus dans l'étude. Les patients non consentants pour l'étude, ceux qui avaient des troubles cognitifs (aphasiques et/ou détérioration cognitive avec MMSE ≤27), les patients perdus de vue après hospitalisation, les patients âgés de moins de 16 ans, les patients dont l'AVC n'avait pu être confirmé par la TDM ou l'IRM encéphalique, n'ont pas été inclus dans l'étude.

Les données des patients ont été collectées prospectivement depuis l'admission en hospitalisation à l'aide de fiches de collecte individuelles par entretien, vérifiées, classées et conservées sous forme de fiches informatiques. Cette collecte a été poursuivie après l'hospitalisation lors de chaque consultation externe de suivi post AVC, le plus souvent tous les 3 mois.

Les variables suivantes ont été prises en compte dans notre étude: caractéristiques sociodémographiques des patients; durée de suivi des patients; caractéristiques cliniques et paracliniques de l'AVC; existence de DCPA et, le cas échéant, caractéristiques cliniques de la DCPA, le type de traitement de la DCPA et son résultat, l'impact de la DCPA sur la qualité de vie des patients. Enfin une comparaison des caractéristiques sociodémographiques, cliniques et neuroradiologiques des patients avec ou sans DCPA a permis d'identifier les variables associées à la survenue de la DCPA, en analyse bivariée, avec un seuil de significativité de p<0,05. Puis les variables avec p<0,20 ont été sélectionnées pour une analyse multivariée avec régression logistique, pas à pas descendante, avec recherche de facteurs de confusion à chaque pas.

Critères diagnostiques obligatoires retenus pour le diagnostic de DCPA:

- douleur dans une zone du corps correspondant à une lésion du système nerveux central;
- antécédents d'AVC avec un début de la douleur survenu après le début de l'AVC;
- confirmation d'une lésion cérébrovasculaire par neuro imagerie (TDM ou IRM encéphalique), associée à des signes sensitifs positifs ou négatifs limités à la zone du corps correspondant à la lésion cérébrovasculaire;
- exclusion des autres causes de douleur, telles que les douleurs nociceptives (notamment mécaniques, inflammatoires ou liées à tout autre dommage tissulaire) ou neuropathiques périphériques;
- score « douleurs neuropathiques en 4 questions » (DN4) ≥ 4 permettant de caractériser la nature neuropathique de la DCPA. Le questionnaire DN4 est un outil diagnostic de conçu et validé par un groupe d'experts français. Ce questionnaire, qui comporte un total de 10 questions, s'appuie exclusivement sur l'interrogatoire des patients et un examen succinct de la sensibilité. Les sept premières questions visent à préciser les caractéristiques de la douleur. Les trois autres questions reposent sur un examen clinique visant à rechercher une hypoesthésie au tact ou à la piqûre et/ou d'une douleur déclenchée par le frottement (allodynie). Un score de 1 est attribué à chaque item positif et le score DN4 total correspond à la somme des réponses aux 10 items. Cet outil permet d'établir le diagnostic de douleur neuropathique avec une spécificité de près de 90% pour un score de 4/10, considéré comme la valeur seuil [3].

La classification de l'intensité de la DCPA a été faite selon l'échelle visuelle analogique (EVA): EVA ≤ 4 douleur de faible intensité; EVA 5-6 douleur d'intensité moyenne; EVA 7-8 douleur très intense; EVA 9-10: douleur intolérable ou insupportable.

L'efficacité du traitement de la DCPA a été jugée par les patients et évaluée par l'amélioration clinique de la douleur selon la réduction de l'EVA dans un délai d'au moins 3 mois après le début du traitement. Le traitement a été jugé peu efficace pour une diminution de l'EVA < 50%, modérément efficace pour une diminution de l'EVA entre 50% et 75% et très efficace pour une diminution de l'EVA > 75%.

L'étude a été approuvée par le Comité national d'éthique du Burkina Faso et a été menée conformément à la Déclaration d'Helsinki.

## Résultats

Du 1^er^ janvier 2015 au 3 mars 2018, nous avons consécutivement colligé 236 patients hospitalisés pour AVC, puis revus tous les 3 mois en consultation externe de neurologie dans le cadre d'un suivi post AVC au CHU de Tingandogo. La durée moyenne de suivi post AVC a été de 12,9 mois ± 10,3 mois (extrêmes 9-39 mois). Durant ce suivi, 28 patients ont présenté des douleurs centrales post AVC (DCPA), soit une prévalence de 11,9%. Parmi les 236 patients suivis pour AVC, il y avait 154 hommes (65,3%) et 82 femmes (34,7%), soit un sex-ratio H/F de 3: cette prédominance masculine (53,6%) était également présente chez les patients qui avaient une DCPA (p = 0,16). La moyenne d'âge de l'ensemble des patients était de 61,6 ans ±14,5 ans (extrêmes 25 ans et 100 ans) alors que ceux qui avaient une DCPA avaient un âge moyen de 54,6 ±14,8 ans (extrêmes 30 - 87 ans) (p ≤ 0,000).

Pour l'ensemble des patients, le score NIHSS moyen lors de l'admission était de 11+/- 5,2 (extrêmes 0 – 28). Il y avait 164 cas d'infarctus cérébral (69,5%), 70 cas d'hémorragie intracérébrale (29,7%) et 2 cas de thrombose veineuse cérébrale (0,8%).

À la fin de l'étude, 87 patients (36,9%) étaient autonomes ou indépendants (score mRS 0-2), 120 patients (50,8%) étaient dépendants pour les activités de la vie quotidienne (mRS 3-5) et 29 patients (12,3%) étaient décédés (mRS 6). Le mRS moyen était de 2,5 +/- 2,1 (extrêmes 0 – 6).

Le délai moyen d'apparition de la DCPA était de 3,8 mois +/- 2,5 (7 jours - 12 mois); la majorité des patients, soit 11 cas (38%), ont vu leur DCPA débuter dans le mois suivant la survenue de l'AVC.

Les douleurs à type de brûlure, avec 21 cas (75%), à type d'allodynies avec 19 patients (68%), à type de froid douloureux avec 15 patients (54%) et à type de décharges électriques avec 14 cas (50%), étaient les types de douleurs les plus fréquemment rencontrés.

L'intensité moyenne de la DCPA était de 7,6 ±1,4 (extrêmes 5 et 10). La douleur était très intense ou sévère (EVA 7 à 8), à très sévère ou insupportable ou intolérable (EVA 9 à 10) chez la majorité des patients, soit 61%.

La localisation de la DCPA était hémicorporelle dans 22 cas (78%), au membre supérieur dans 4 cas (14%) et au membre inférieur dans 2 cas (7%).

La douleur était permanente chez 22 patients (81%) et intermittente chez 6 patients (18%).

Les facteurs déclenchants de la douleur étaient dominés par le frottement (71%), la pression et l'émotion, respectivement dans 61% chacun.

Les facteurs calmant de la douleur étaient représentés par le repos, la relaxation et le massage, respectivement chez 21 patients (75%), 8 patients (28%) et 4 patients (14%).

Une hypoesthésie algique et au tact grossier (homolatérale à la douleur) était présente chez 92 patients sur l'ensemble des 236 patients (39%) et chez 27 patients avec DCPA (96%) et 70 patients sans DCPA (33,6%) (p = 0,036). Chez les patients avec DCPA, l'hypoesthésie était tactile (tact grossier) ou algique, isolée ou associée, chacune chez 19 patients (68%).

Lors de l'apparition de la DCPA, 18 patients sur les 28 patients avec DCPA, soit 64%, avaient une régression notable du déficit neurologique. Le tableau I ci-après présente la répartition des patients selon l'évolution du déficit neurologique lors l'apparition de la DCPA. Les paresthésies à type d'engourdissement avec 20 cas (71%), de fourmillements avec 13 cas (46%), isolés ou associés, étaient les symptômes les plus fréquemment associés à la DCPA. Le tableau II présente la répartition des patients selon les symptômes d'accompagnement de la DCPA.

**Tableau I:** Répartition des patients selon l'évolution du déficit neurologique lors l'apparition de la douleur (n = 28) Distribution of patients according to the evolution of the neurological deficit at the onset of pain (n = 28)

Évolutions clinique du déficit neurologique	Effectif	%
aggravation	1	4
régression notable	18	64
stationnaire	9	32
total	28	100

**Tableau II:** Répartition des patients selon les symptômes d'accompagnement de la DCPA Distribution of patients according to symptoms accompanying CPSP

	Effectifs (n)	%
Symptômes d'accompagnement de la DCPA		
démangeaisons	12	43
engourdissement	20	71
picotements	6	21
fourmillements	13	46
douleur provoquée ou augmentée au frottement	17	61

La DCPA avait un retentissement négatif modéré à sévère sur le travail habituel chez 61% des patients, sur l'activité générale chez 50% des patients, sur l'humeur chez 46% des patients, sur les capacités physiques chez 39% des patients, sur le sommeil chez 36%, sur les interactions sociales chez 21% des patients et sur le goût de vivre chez 11% des patients.

L'amitriptyline avec 21 cas (75%) et les antalgiques de palier II avec 17 cas (61%), ont été les molécules les plus fréquemment utilisées dans la prise en charge de la DCPA, le plus souvent en bi ou trithérapie. Le tableau III présente la répartition des patients selon les molécules utilisées pour le traitement de la DCPA.

**Tableau III:** Répartition des patients selon les molécules utilisées pour le traitement de la DCPA Distribution of patients according to molecules used to treat CPSP

Molécules		Effectifs (n)	%
**Antalgiques de palier II**		25	89
tramadol		16	64
paracétamol + codéine		9	32
**Antiépileptiques** (gabapentine ou prégabaline)		14	50
**Antidépresseurs**		16	64
antidépresseurs tricycliques (amitriptyline)		13	46
inhibiteurs de la recapture de la sérotonine et noradrénanaline		3	11

Le traitement médicamenteux reçu a été jugé moyennement efficace à très efficace chez 15 patients (57%), peu efficace chez patients (32%) et totalement inefficace chez 3 patients (11%).

Après analyse bivariée entre les caractéristiques sociodémographiques, cliniques et neuroradiologiques (variables indépendantes) et la survenue de la DCPA (variables dépendantes), l'âge ≤ 50 ans (p = 0,001) et la présence d'une hémi-hypoesthésie (p = 0,035), étaient significativement associés à la survenue de la DCPA. Le tableau IV, présente les résultats de l'analyse bivariée entre les caractéristiques sociodémographiques, cliniques et radiologiques (variables indépendantes) et la survenue de la DCPA (variable dépendante).

**Tableau IV:** Résultats de l'analyse bivariée entre les caractéristiques sociodémographiques, cliniques et neuroradiologiques (variables indépendantes) et la survenue de la DCPA (variables dépendantes) Results of bivariate analysis between sociodemographic, clinical and neuroradiological characteristics (independent variables) and CPSP occurrence (dependent variables)

Variables	Douleur		OR [95% IC]	P
	n (%)	n (%)		
**Genre**	DCPA +	DCPA -		
féminin	13 (15,85)	69(84,15)	1,74[0,78-3,87]	0,16
masculin	15 (9,74)	139(90,26)		
**Âge**				
≤ 50	12 (26,09)	34(73,91)	3,83[1, 66-8, 83]	0,00008
> 50	16 (8,42)	174(91,58)		
**HTA**				
oui	21 (11,73)	158(88,27)	0,94[0,38-2,36]	0,91
non	7 (12,28)	50 (87,72)		
**Diabète**				
oui	4 (16)	21(84)	1,48 [0,46-4,69]	0,49
non	24 (11,37)	187(83,63)		
**Tabagisme**				
oui	1 (8,33)	11 (97,67)	0,66 [0, 085, 34]	0,69
non	27 (12,05)	197(87,95)		
**Gradation mRS**				
mRS ≤ 3	18(12,86)	122(87,14)	1,26 [0,55-2,88]	0,56
mRS > 3	10 (10,42)	86 (89,58)		
**Nature AVC**				
infarctus	17 (10,37)	147(89,63)	0,64 [0,28-1,44]	0,28
HIC/TVC	11 (15,28)	61 (84,72)		
**Déficit sensitif objectif**				
oui	27 (13,92)	167(86,08)	6,62[0,87-50,21]	0,036
non	1 (2,38)	41 (97,62)		

Après analyse multivariée avec régression logistique pas à pas, seul l'âge ≤ 50 ans était indépendamment associé à la survenue de la DCPA (OR 2, 86; 95% IC [1,09-7,46]; p = 0,03).

## Discussion

Les limites de notre étude tiennent au caractère non homogène de la durée du suivi post AVC des patients qui a pu faire sous-estimer la DCPA et au caractère hospitalier de l'étude avec une tendance au biais de sélection des patients les plus souffrants, qui a pu contribuer à surestimer l'incidence de la DCPA. Le caractère hospitalier de notre étude, ne permet pas l'extrapolation de nos résultats à toute la population.

Dans notre étude, nous avons retrouvé une prévalence de la DCPA de 11,9%, qui s'inscrit dans une large fourchette de prévalence variant de 1 à 12% dans la grande majorité des données de la littérature [15, 21, 22, 23, 24, 41]. Ainsi, la prévalence rapportée dans notre étude, se rapproche de celle de 11,8% rapportée au Danemark [28], de 11% respectivement rapportée au Royaume-Uni [4] et en Italie [41] et de 12% rapportée à Singapour [25]. Par contre, elle est nettement plus élevée que celles rapportées en Suède [33], au Nigéria [2, 38] et en Finlande [17], qui étaient respectivement de 3%, 5 à 8% et 5,9%. Certains auteurs ont toutefois rapporté des prévalences plus élevées, de l'ordre de 20,7% en Inde [29], entre 8 à 35% en Croatie [34] et de l'ordre de 35% en Suède [48]. Cette différence de prévalence de la DCPA selon les études, est due aux variations des critères d'inclusion, des critères de définition de la DCPA et de la durée des études, du fait de l'extrême complexité des caractéristiques cliniques, des difficultés à distinguer ce syndrome des autres types de douleur post AVC et à l'absence de critères diagnostiques pathognomoniques, notamment l'absence de marqueurs biologiques pour la DCPA [30].

Le délai d'apparition de la DCPA est variable selon les données de la littérature [24, 37], soit immédiat après l'AVC, éventualité cependant plus rare [4, 41], ou le plus souvent dans le mois voire plusieurs années après l'AVC [4, 24, 27, 28, 37]. Dans notre série, le délai d'apparition de la DCPA variait entre une semaine à 3 mois après l'AVC, chez 50% des patients: assez proche des autres séries de la littérature. Cette variation du délai d'apparition de la DCPA interpelle les praticiens sur la vigilance et l'attention qu'ils doivent accorder aux symptômes ou signes de la DCPA lors du suivi post AVC des patients, afin de poser un diagnostic précoce et d'entreprendre un traitement adéquat, important pour l'amélioration de la qualité de vie des patients. Le fait que le CPSP soit plus fréquent dans les stades subaigus et chroniques est conforme aux études précédentes montrant que la douleur neuropathique, probablement en raison de modifications plastiques des voies somatosensorielles et de la douleur, est une complication retardée après un AVC.

Les caractéristiques cliniques principales de la DCPA fréquemment décrites dans la littérature par les patients étaient les sensations de brûlure, de décharges électriques, de froid douloureux, et les paresthésies à type de fourmillements, de picotements, et les sensations d'engourdissement [2, 10, 14, 32]. En effet, les sensations de brûlures étaient le symptôme le plus fréquemment rapporté, respectivement chez 75% des patients de notre étude, 62% des patients au Nigéria [2], 57% des patients au Royaume-Uni [4] et 49,2% des patients en Italie [39].

L'intensité et le rythme de la DCPA sont diversement rapportés dans la littérature selon l'EVA, allant d'une douleur minime peu intense (EVA ≤ 4) ou une douleur moyennement intense (EVA 5 et 6), intermittente, à une douleur sévère ou très intense (EVA 7 et 8) voire très sévère ou insupportable (intolérable), permanente ou intermittente [19]. Dans notre série, le profil d'intensité était en moyenne celui d'une douleur très intense (EVA de 7,6 ± 1,4); la douleur était très intense à insupportable dans 61% et elle était permanente dans 81,5% des cas. Nos résultats sont ainsi conformes aux données de la littérature. Les importantes variations d'intensité de la DCPA observées à travers les études reflètent le caractère subjectif de la douleur et les difficultés d'utilisation des outils d'évaluation de cette douleur.

Parmi les facteurs déclenchants de la DCPA, les plus fréquemment cités sont les stimuli mécaniques (mouvement, toucher), ou thermiques (particulièrement le froid) [4, 24, 48], suivis des émotions et du stress [9, 29]. Parmi les facteurs calmants, la relaxation a été souvent rapportée [4]. Ces facteurs déclenchants et calmants ont été diversement retrouvés dans notre étude, tout comme dans une série du Nigeria [2].

Dans notre série, dans 50% des cas, la DCPA apparaissait chez des patients qui avaient une régression notable du déficit neurologique: ce qui est en accord avec les données de la littérature, mentionnant que la DCPA survient en général chez des patients peu déficitaires [4], ou lors de la récupération ou après une amélioration partielle du déficit neurologique initial [29].

En réduisant les capacités physiques, les performances sociales et le bien-être psychologique des patients, la DCPA a un impact négatif sur la qualité de vie des patients en post AVC, selon les données de la littérature [15, 17, 18, 26, 47]. La DCPA peut également conduire à la dépression, à l'anxiété, à des troubles du sommeil [16, 29, 31], à une perte d'appétit, à une dépendance à la drogue, à de mauvaises interactions sociales et contribue à réduire les chances de récupération fonctionnelle et de reprise de l'activité professionnelle [16, 31]. À terme, une DCPA réfractaire au traitement peut provoquer une dépression sévère, pouvant conduire au suicide [11] ou à des automutilations [37]. Avec un impact global négatif de la DCPA sur la qualité de vie de nos patients, notre étude est en accord avec les données de la littérature. En effet, la DCPA interférait négativement sur plusieurs aspects de la qualité de vie de nos patients: les plus fréquemment impactés étaient les activités professionnelles (60,7%), l'activité générale (50%), l'humeur (46,4%), les capacités physiques (39,3%) et le sommeil (35,7%).

Les patients atteints de DCPA répondent mal aux traitements analgésiques conventionnels [36, 40]. En général, les antidépresseurs, les antiépileptiques et les opioïdes sont utilisés, souvent en combinaison [42]. Cependant, l'efficacité de ces médicaments est discutable en raison d'un manque de grands essais contrôlés [6]. Dans notre étude, les opioïdes faibles (tramadol ou codéine) ont été largement employés chez 89,3% des patients, suivis des antiépileptiques (gabapentine ou prégabaline) chez 50% des patients et les antidépresseurs (tricycliques ou IRSN) chez 64% des patients, le plus souvent en bithérapie. Ce qui est en accord avec les données de la littérature. Chez les patients ne répondant pas aux traitements médicamenteux, la stimulation cérébrale profonde par électrodes implantées, s'est révélée efficace et peut donc être discutée chez certains patients. Des études plus récentes ont démontré l'efficacité de la stimulation magnétique transcrânienne répétitive (rTMS) au niveau du cortex moteur. [20]. Nous n'avons cependant pas pu utiliser ces méthodes thérapeutiques non encore disponibles dans notre contexte de travail.

Selon la plupart des données de la littérature, la réponse au traitement médicamenteux de la DCPA est habituellement modérée, et la posologie est limitée par les effets secondaires, en particulier chez les patients âgés [8, 12, 24, 44, 45]. Ce même constat a été fait dans notre étude. En effet, le résultat du traitement a été jugé moyennement efficace chez 50% et très efficace chez seulement 7% de nos patients. Ce résultat modeste est en partie dû aux effets secondaires, obligeant soit à s'en tenir à des doses infra thérapeutiques (les doses recommandées étant de 75 mg pour l'amitriptyline, 300 à 600 mg pour la prégabaline et jusqu'à 3000 mg pour la gabapentine), soit à l'arrêt prématuré du traitement.

Dans notre série, l'âge ≤ 50 ans a été identifié comme la seule variable indépendamment associée à la survenue de la DCPA (OR 2,86; p =0,03). Le même constat a été dans d'autres séries [17, 35, 39, 46], alors que pour beaucoup d'autres auteurs, le risque de développer une DCPA n'était lié ni à l'âge, ni au sexe, ni au type d'AVC ou à la localisation lésionnelle [1, 24, 33]. Les patients d'âge ≤ 50 ans ont un plus grand risque de développer une DCPA, comparativement à ceux plus âgés, du fait d'une probable plus grande susceptibilité des personnes jeunes à la douleur, d'un pronostic fonctionnel et vital habituellement meilleur chez les patients jeunes en post AVC, d'attentes plus drastiques en termes de qualité de vie, y compris une vie sans douleur en post AVC.

## Conclusion

Notre étude en milieu hospitalier a montré que la DCPA touche plus d'un patient sur 10 victimes d'AVC, le plus souvent jeunes et qu'elle apparait majoritairement dans le mois suivant l'AVC. Elle se caractérise le plus souvent par une sensation de brûlure et/ou d'allodynies hémicorporelles, très intenses à insupportables dans la majorité des cas, et s'accompagne fréquemment d'une hypoesthésie algique et/ou tactile et de paresthésies, de même topographie. Le traitement moyennement efficace, repose majoritairement sur l'amitriptyline et la prégabaline/gabapentine. La DCPA impacte négativement de façon modérée à sévère la qualité de vie des patients, notamment l'activité générale, l'humeur, le sommeil… L'âge ≤ 50 ans constitue un facteur de risque de survenue de la DCPA, du fait probablement d'une plus grande susceptibilité de cette tranche d'âge.

Le dépistage et la prise en charge adéquate de la DCPA dans le cadre du suivi pluridisciplinaire post AVC, contribuent à l'amélioration de la qualité de vie des patients victimes d'AVC et facilitent leur réinsertion socioprofessionnelle.

## Conflits D'intérêts

Les auteurs ne déclarent aucun conflit d'intérêts.
